# A Rare Case of Glioblastoma Multiforme with Osseous Metastases

**DOI:** 10.1155/2017/2938319

**Published:** 2017-10-19

**Authors:** Rubens Barros Costa, Ricardo Costa, Jason Kaplan, Marcelo Rocha Cruz, Hiral Shah, Maria Matsangou, Benedito Carneiro

**Affiliations:** Developmental Therapeutics Program, Feinberg School of Medicine and Robert H. Lurie Comprehensive Cancer Center of Northwestern University, 233 East Superior Street, Chicago, IL 60611, USA

## Abstract

Glioblastoma multiforme is the most common malignant primary central nervous system neoplasm in adults. It has a very aggressive natural history with a median overall survival estimated at 14.6 months despite multimodality treatment. Extracranial metastases are very rare with few case reports published to date. We report the case of a 65-year-old male who underwent maximal safe resection for a newly diagnosed brain mass after presentation with new neurologic symptoms. He then received standard postsurgical adjuvant treatment for glioblastoma. Subsequently, he underwent another resection for early progressive disease. Several months later, he was hospitalized for new-onset musculoskeletal complaints. Additional investigation revealed new metastatic osseous lesions which were initially felt to be a new malignancy. The patient opted for supportive care and died 12 days later. Despite choosing no treatment, he elected to undergo a bone biopsy to understand the new underlying process. Results were that of metastatic GBM and were reported after the patient expired. Physicians caring for patients with GBM and new nonneurologic symptoms may contemplate body imaging.

## 1. Introduction

Glioblastoma multiforme (GBM) is a common primary central nervous system (CNS) neoplasm in adults with a very aggressive natural history and grim prognosis [1, 2]. For fit patients, the standard treatment approach is maximal safe resection with adjuvant brain radiation and temozolomide. Important prognostic factors are age and performance status. The median overall survival is estimated at 14.6 months with combined therapy [3]. The cause of death is usually related to local progression and its complications. Extracranial metastases are rare, with reports scarcely available. Possible explanations for the lack of metastatic dissemination are short survival time and the presence of the blood brain barrier. For these reasons, screening for metastases is not a common practice. The median survival time from metastasis diagnosis is short (∼14 months) [3–5]. Herein, we report a case of glioblastoma multiforme with metastases to the bones and a poor outcome.

## 2. Case Report

**Figure 1:**
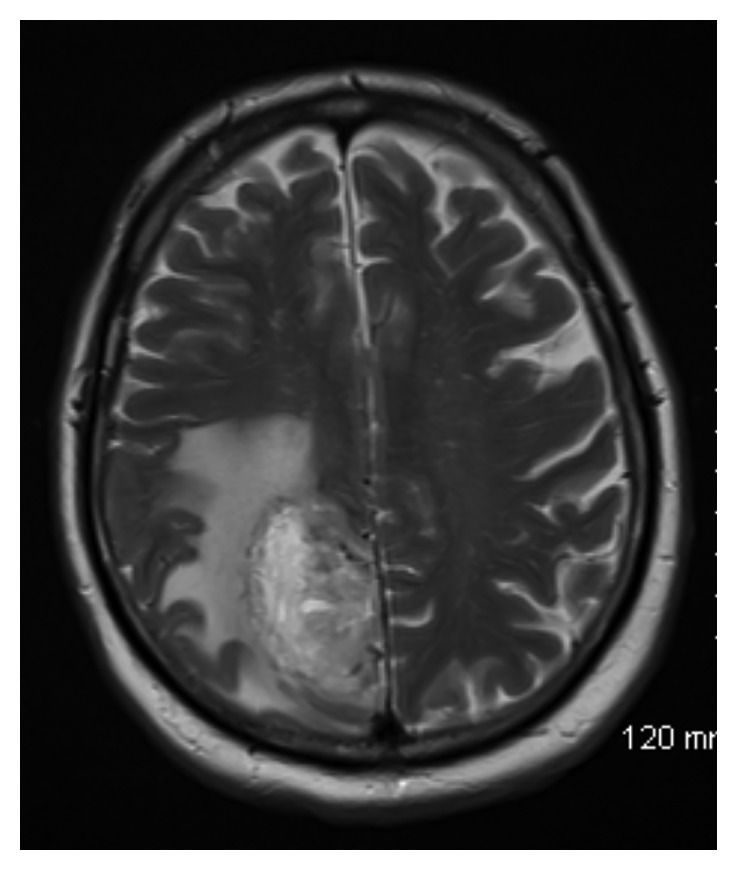
Axial T2-weighted image demonstrating heterogeneous mass in the right parasagittal parietal lobe with extensive surrounding vasogenic edema.

The patient was a 65-year-old gentleman with a past medical history of hypertension and diabetes mellitus type 2 who was admitted to the hospital with progressive weakness, headache, dizziness, and confusion. His general and neurologic exam was grossly normal. Blood laboratory data were within normal limits. A nonenhanced computed tomography (CT) of the brain showed a large mass within the right parietal lobe with surrounding vasogenic edema. A brain magnetic resonance imaging (MRI) showed an irregularly enhancing mass measuring 5.8 cm anteroposterior × 3.2 cm transverse × 4.4 cm craniocaudal within the right parieto-occipital lobe, with significant vasogenic edema (Figures 1() and 2()). He then underwent a right parietal craniotomy with maximal safe resection of the tumor.

**Figure 3:**
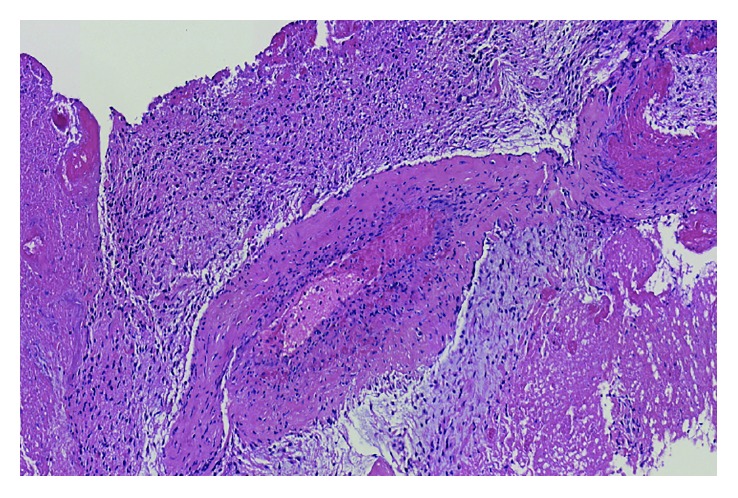
Brain surgical specimen. High-grade glioma with foci of necrosis and microvascular proliferation consistent with glioblastoma multiforme (WHO Grade IV).

The surgical pathology report showed a high-grade glioma with foci of necrosis and microvascular proliferation consistent with GBM (WHO Grade IV) (Figure 3()). His postsurgical course unfolded without complications.

He then went on to receive adjuvant radiation therapy with a total of 60 Grays divided into 30 fractions during the course of 44 days. He additionally received temozolomide at 75 mg/m^2^ daily while on radiation therapy, with essentially no toxicity. Temozolomide was subsequently continued at 150 mg/m^2^ daily five days out of a 28-day cycle.

A follow-up MRI of the brain approximately 5 weeks post completion of combined therapy showed increased enhancement and diameter of the parietal mass, mass effect on the adjacent right occipital horn of the right ventricle, and diffuse vasogenic edema on the right cerebral hemisphere.

Subsequently, the patient was transferred to an outlying institution for additional management where he underwent reopening craniotomy with microsurgical resection of the tumor with Gliadel wafer placement approximately 17 weeks from his initial resection. The surgical specimen showed progressive glioblastoma. His postsurgical recovery was complicated by hydrocephalus, hemiparesis, and an acute non-segment elevated myocardial infarction. He returned to the operating room for a right ventriculoperitoneal shunt placement on postoperative day 12.

About 4 weeks after the procedure, the patient was started on bevacizumab every 3 weeks. He received 5 treatments with this agent which had to be discontinued due to proteinuria and hypertension. A follow-up brain MRI five months post second resection showed postoperative changes without evidence of recurrent and/or progressive disease.

**Figure 4:**
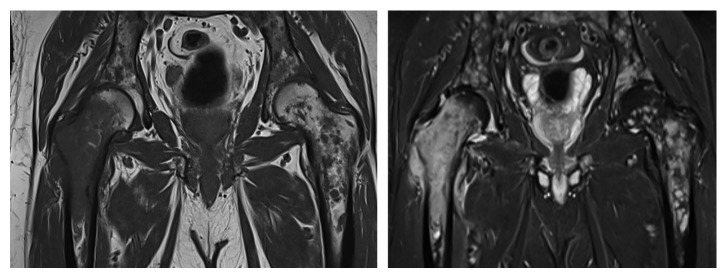
Coronal T1- (left) and T2- (right) weighted images approximately 6 months following resection demonstrating extensive neoplastic lesions throughout both femurs, iliac bones, and the sacrum.

**Figure 5:**
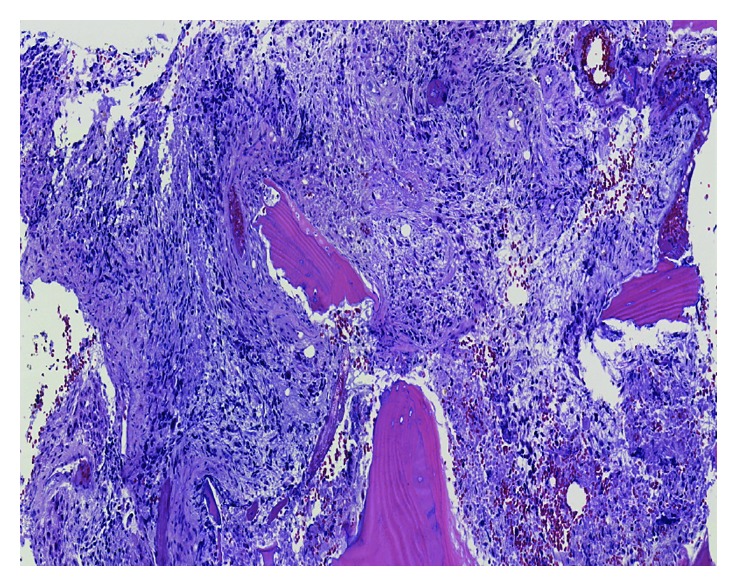
GBM osseous metastasis (higher power). Microscopic sections showing a destructive cellular neoplasm with highly atypical and hyperchromatic tumor cells, abundant necrosis, and vascular proliferation.

**Figure 6:**
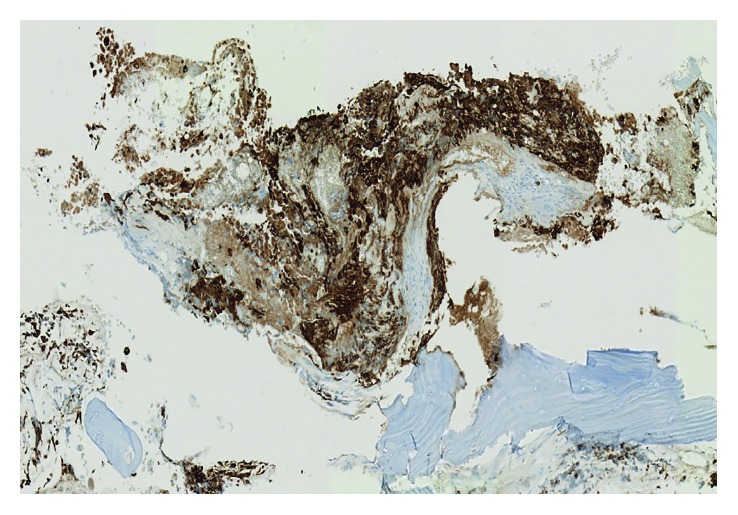
GBM with GFAP stain. GFAP (glial fibrillary acidic protein) immunohistochemical stain showing strong staining within the fibrillary cytoplasmic processes of the viable tumor cells.

Approximately five weeks after his most recent follow-up MRI, he was hospitalized with excruciating lower back pain radiating to his right hip and anterior thigh. MRI of the pelvis showed extensive lesions with involvement of femurs, iliac bones, and sacrum (Figure 4()). A CT chest, abdomen, and pelvis was negative for visceral metastatic disease. Bone scan (Tc 99m) showed subtle uptake in the bilateral humeri and increased uptake in the region of the greater trochanter on the right. Subtle increased uptake in the trochanteric region on the left was also reported. A biopsy of a right femoral lesion showed numerous tumor cells in a reactive stroma replacing much of the bone marrow (Figure 5()). The tumor cells stained positive for glial fibrillary acidic protein (GFAP) (Figure 6()). The conclusion was that it was representative of a high-grade astrocytoma (i.e., glioblastoma). Given the limited therapeutic options and poor performance status, the patient elected for supportive care only and died 12 days after the biopsy. No brain imaging was performed during the hospitalization.

## 3. Discussion

GBM is a very common and aggressive malignant neoplasm of the CNS in adults. In the seminal paper by Stupp et al., the median overall survival was roughly 14.6 months for patients who underwent resection, followed by adjuvant chemoradiotherapy [3]. With an estimated 5-year survival rate of less than 5%, the cause of death is almost always related to local progression and its complication. Osseous and visceral metastases are a rare phenomenon in the natural history of GBM with incidences reported anywhere from 0.4 to 2% [4]. Possible explanations for the low occurrence or detection of extracranial dissemination are short survival and the blood brain barrier [4]. It is possible that metastasis is not a rare occurrence, but for most patients it will never represent a life-threatening event in light of almost universal early progression in the CNS. For this reason, clinicians usually do not screen for disease spread outside of the brain. Indeed, the most recent version of the National Comprehensive Cancer Network Guidelines does not have a formal recommendation to screen for metastases in patients with high-grade gliomas after maximal safe resection [6].

A recent review by Kalokhe et al. found 79 cases with extracranial disease for patients who were 18 years or older, proven by either biopsy or autopsy. The most common sites of metastases were the bone (38%), lymph nodes (37%), lungs (32%), and liver (18%). Other organs included the eyes, spleen, liver, parotid and adrenal glands, and subcutaneous tissues [4]. Malignant effusion may also be the initial presentation of GBM in patients without evidence of brain pathology on brain imaging after solid organ allografting from a previous donor with GBM found on autopsy [7]. In this analysis, the median survival in their analysis was 13 months compared to 14 months in the seminal trial by Stupp. The median survival from the time of diagnosis of metastatic disease was 5 months. Chemotherapy but not surgery and/or radiation was the only therapeutic modality that possibly lengthened survival and time to metastases, barring the limitations of their report [4].

More recently, Fonkem et al. have pointed out that GBM cells may be present in the circulation as there is evidence that the blood brain barrier may be disrupted in GBM [8, 9]. Furthermore, another report by Franceschi et al. showed mutations in patient blood germinal DNA that matched that of a primary GBM with biopsy-proven metastasis to the sternum [10]. This is important in that it could allow for accrual of patients with very few treatment options into multiple-histology basket trials while circumventing the need for invasive biopsies. Nonetheless, they also postulated that the immune system may suppress the growth of GBM cells in the circulation, preventing its seeding in other organs. Building upon this concept, other cases of extracranial GBM metastases in recipients of donors of solid organ with previously treated GBM have been described [8, 11–13]. Further understanding of mechanisms of immune evasion leading to disease progression could allow for possible use of cytotoxic immune stimulation with checkpoint inhibitors as is in other solid tumors.

This report adds to others of previous metastatic GBM [4, 10, 11, 14, 15]. Our patient faired very poorly, in keeping with the other cases in the literature. His survival post diagnosis was approximately 8 months. We cannot be certain of his cranial disease status at time of diagnosis of metastatic disease. We could possibly speculate that the placement of the ventriculoperitoneal shunt may have contributed to disseminated metastasis as others have previously as a mechanism of spread of a primary CNS tumor [16].

In summary, this case illustrates that extracranial metastatic disease may be an event in the natural course of the patient's GBM.

## Figures and Tables

**Figure 2:**
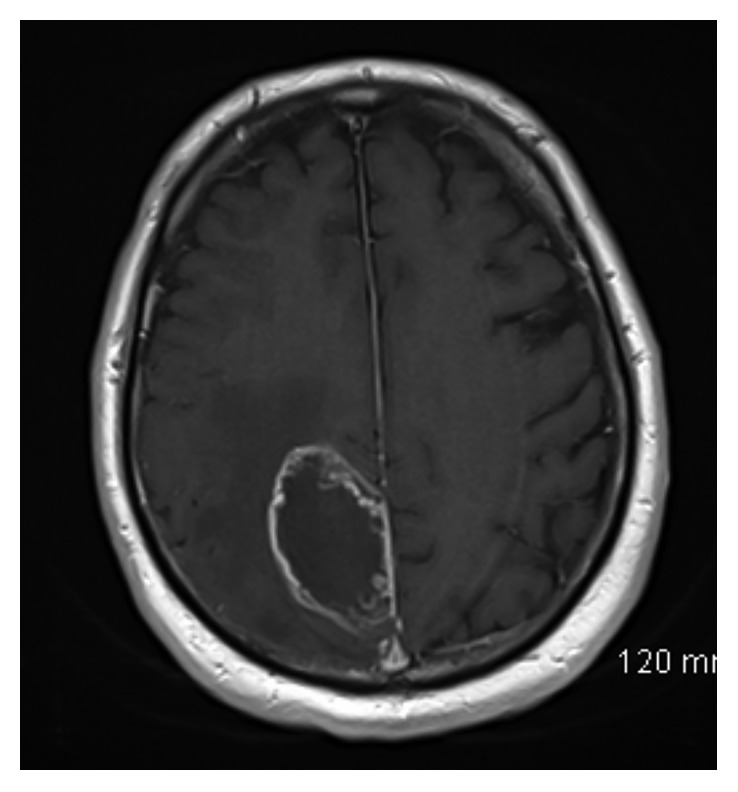
Axial T1-weighted image after administration of gadolinium demonstrating peripheral nodular enhancement of right parietal mass.
